# Intranasal vaccination with a recombinant protein CTA1-DD-RBF protects mice against hRSV infection

**DOI:** 10.1038/s41598-021-97535-6

**Published:** 2021-09-20

**Authors:** Hai Li, Hu Ren, Yan Zhang, Lei Cao, Wenbo Xu

**Affiliations:** 1 NHC Key Laboratory of Medical Virology and Viral Diseases, National Institute for Viral Disease Control and Prevention, Chinese Center for Disease Control and Prevention, No. 155, Changbai Road, Changping District, Beijing, 102206 People’s Republic of China; 2 Center for Biosafety Mega-Science, Chinese Academy of Sciences, Beijing, People’s Republic of China

**Keywords:** Infectious diseases, Vaccines

## Abstract

Human respiratory syncytial virus (hRSV) infection is a major pediatric health concern worldwide. Despite more than half a century of efforts, there is still no commercially available vaccine. In this study, we constructed and purified the recombinant protein CTA1-DD-RBF composed of a CTA1-DD mucosal adjuvant and prefusion F protein (RBF) using *Escherichia coli* BL21 cells. We studied the immunogenicity of CTA1-DD-RBF in mice. Intranasal immunization with CTA1-DD-RBF stimulated hRSV F-specific IgG1, IgG2a, sIgA, and neutralizing antibodies as well as T cell immunity without inducing lung immunopathology upon hRSV challenge. Moreover, the protective immunity of CTA1-DD-RBF was superior to that of the RBF protein, as confirmed by the assessment of serum-neutralizing activity and viral clearance after challenge. Compared to formalin-inactivated hRSV (FI-RSV), intranasal immunization with CTA1-DD-RBF induced a Th1 immune response. In summary, intranasal immunization with CTA1-DD-RBF is safe and effective in mice. Therefore, CTA1-DD-RBF represents a potential mucosal vaccine candidate for the prevention of human infection with hRSV.

## Introduction

Human respiratory syncytial virus (hRSV) is an orthopneumovirus belonging to the Pneumoviridae family^1^. Since its identification in children with pneumonia 60 years ago^2^, hRSV has been established as an important cause of acute lower respiratory illness (ALRI) in infants and children worldwide^3^, and it also infects elderly and immunocompromised individuals. Despite more than half a century of efforts, there is still no commercially available vaccine^4^. Prophylaxis with the humanized monoclonal antibody palivizumab is the only viable intervention for hRSV, but it is limited to use in high-risk infants due to its modest efficacy and high cost^5^. In recent years, progress on elucidating the structural biology of the hRSV fusion glycoprotein (F) has provided new directions for the development of hRSV vaccines, and more hRSV candidate vaccines that utilize different technologies and target different populations are rapidly being developed^6^. More than 60 kinds of hRSV candidate vaccines are being assessed for infants and elderly individuals in particular, and most are in the preclinical stage^7,8^.

The highly conserved F protein can induce antibodies against infections caused by hRSV of both subgroups A and B^9^ and is therefore a key target in the development of subunit vaccines, particle-like vaccines and viral vector-based vaccines^10–12^. The F protein is initially expressed as an uncleaved F0 precursor that is activated by furin cleavage at two sites into the mature prefusion F protein (pre-F)^13,14^. The F protein is a trimeric glycoprotein that transitions between a pre-F conformation and a postfusion structure to facilitate hRSV entry into target cells^15,16^. Pre-F is metastable and spontaneously rearranges into a highly stable postfusion state^17^. McLellan et al. identified that the pre-F protein with epitope zero (Ø) (aa 62–69, aa 196–209) located at the apex of the trimeric pre-F elicits more neutralizing activity in mice than the post-F protein^18^. The hRSV-neutralizing antibodies AM22, D25 and 5C4 (specific to prefusion F protein) have been found to be substantially more potent than palivizumab (which binds both the pre-F and post-F proteins)^18,19^. After the discovery of methods to stabilize the F protein in its pre-F conformation, numerous pre-F candidate vaccines have been developed^20–22^, and the Ø site is a desired characteristic of next-generation hRSV F antigens^23^.

hRSV infects via mucosal surfaces, and low levels of hRSV-specific nasal sIgA were found to be an increased risk factor for hRSV infection^24^. Inducing immunity via the noninvasive mucosal route has been explored to induce mucosal antibody responses and does not cause enhance respiratory disease (ERD)^25^. Therefore, some researchers have focused on mucosal immunity in preclinical studies and clinical trials^26–28^. All live attenuated vaccines that enter clinical trials are administered through the nose^29–32^, and viral vector vaccines administered nasally, including PanAd3-RSV, MVA-RSV^33^ and SeVRSV^34^, have also shown good safety and efficacy in adults. The successful development of a mucosal vaccine depends on the use of safe and effective adjuvants.

The nontoxic mucosal adjuvant CTA1-DD comprises the CTA1 subunit of cholera toxin (CT) and two immunoglobulin (Ig) binding domains (DD) of staphylococcal protein A (SpA). CTA1 is an adenosine diphosphate (ADP)-ribosyltransferase^35^. SpA contains five highly homologous extracellular domains in tandem, designated domains E, D, A, B, and C. The domain D consist of three a-helical structures^36^. CTA1-DD has been shown to bind to all immunoglobulins through the DD domain, including Ig on the B cell surface^37^. To date, the CTA1-DD mucosal adjuvant has been demonstrated to be effective against many pathogens, including Ebola virus, influenza virus, and HIV^38–40^. Combining the neutralizing peptides and CTA1-DD into one protein confers strong protective immunity against *Helicobacter pylori* and influenza virus in mice^41,42^. A substantial amount of preclinical data have confirmed the efficiency and safety of CTA1-DD, which is one of the most promising mucosal adjuvants to date^43^. Although previous research suggests that the F-ctxA2B fusion protein consists of residues 412–524 of the hRSV F protein and that the ctxA2B subunit of CT can provide partial protection^44^, no research has been performed on the CTA1-DD mucosal adjuvant used in hRSV vaccines until now.

In the present study, we constructed and purified the CTA1-DD-RBF protein using *E. coli* BL21 and tested its immunogenicity and protective efficacy in mice. Compared with RBF, CTA1-DD-RBF induced higher levels of neutralizing serum antibodies and inhibited virus replication in the lungs of immunized mice. Intranasal vaccination induced a Th1-biased immune response, while formalin-inactivated RSV (FI-RSV) induced Th2-biased immune responses. This study demonstrates that the uncleaved CTA1-DD-RBF protein expressed in *E. coli* elicits protective immunity in mice without enhancing the disease.

## Results

### Production, purification and conformation of the CTA1-DD-RBF protein

**Figure 1:**
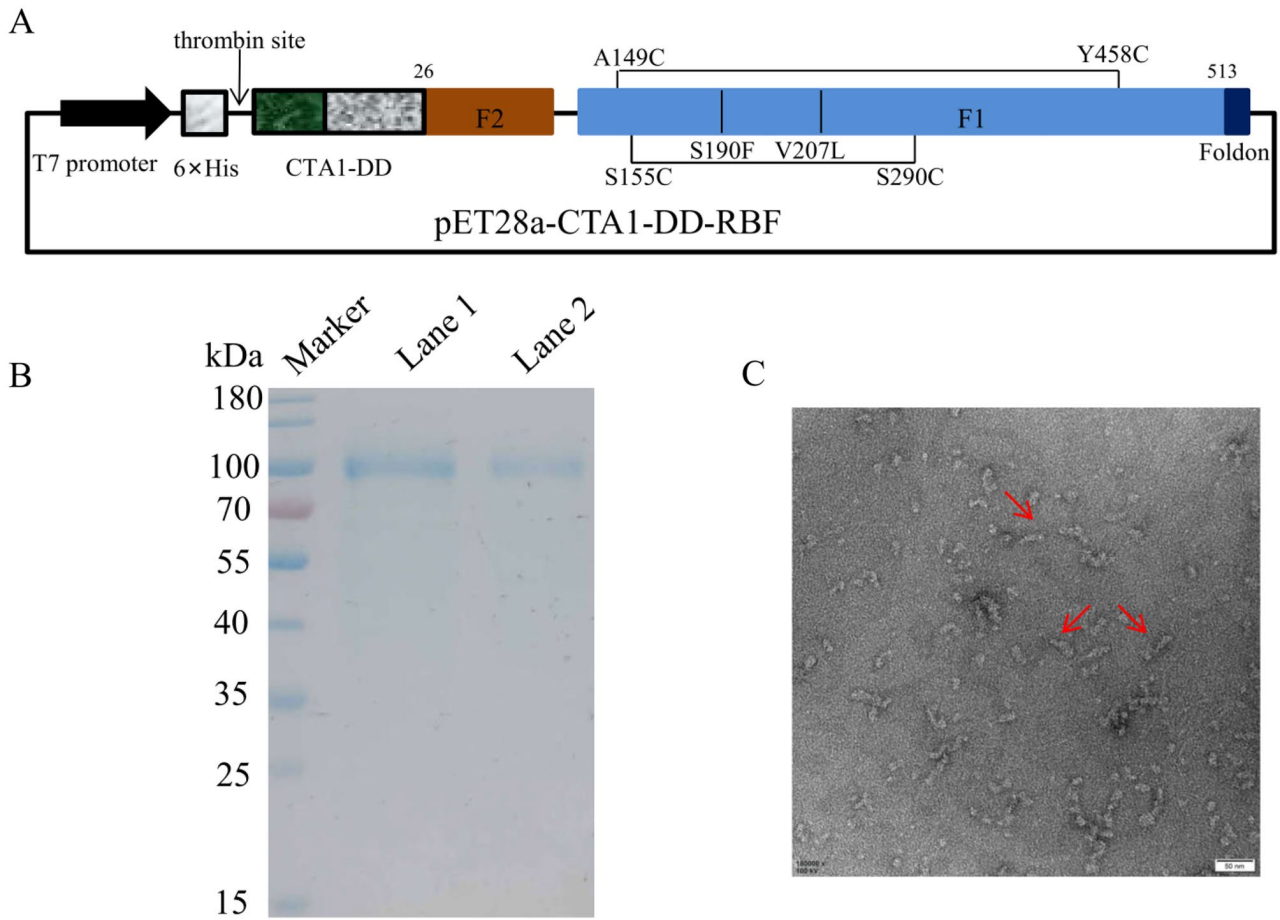
Design and analysis of the CTA1-DD-RBF protein. (**A**) Domain structure of the CTA1-DD-RBF protein. Mutations (Val to Leu at residue 207 and Ser to Phe mutation at residue 190; disulfide between residues 290 and 155) and disulfide bonds 149 to 458 were incorporated into the F protein and are indicated by vertical black lines. CTA1-DD-RBF contains the CTA1 subunit of cholera toxin (CT), two immunoglobulin (Ig) binding domains (DD) of staphylococcal protein A and hRSV F protein (residues 26–105 and 146–513) with a T4 fibritin trimerization motif (foldon) and a variable linker, GSGSG. (**B**) CTA1-DD-RBF proteins digested by thrombin on SDS-PAGE: Marker: protein markers; Lane 1: CTA1-DD-RBF purified using a HisTrap FF column after digested by thrombin; Lane 2, CTA1-DD-RBF purified by HisTrap FF columns before digested by thrombin. (**C**) Negative stain electron microscopy of CTA1-DD-RBF. The CTA1-DD-RBF was highly homogenous. Bar = 50 nm.

CTA1-DD-RBF was purified by HisTrap FF columns, and the tags were removed by overnight digestion with thrombin at 26 °C. The protein (without a 6 × His-tag) was then further purified using a Superdex 200 column. To verify the efficacy of thrombin cleavage, CTA1-DD-RBF was further purified using a HisTrap FF column after digestion by thrombin. SDS-PAGE analysis indicated that the purified CTA1-DD-RBF (the protein that passed through the HisTrap FF column) was ≥ 90% pure, and a 90 kDa band was identified (Fig. 1B). Nearly 60% of the CTA1-DD-RBF proteins passed through the HisTrap FF column after digestion by thrombin, indicating that the efficacy of thrombin cleavage was high. The thrombin site is native to the pET28a plasmid, and thrombin cleaves and removes 17 amino acids, including the 6 × His-tag. However, 17 amino acids remained on the CTA1-DD-RBF protein. After chromatographic purification and ultrafiltration, the endotoxin concentration was 4.22 EU per mg CTA1-DD-RBF.

The binding kinetics of CTA1-DD-RBF, RBF and Post-RBF with 5C4 were identified by BLI on the Octet RED96e instrument. The KD (M) for CTA1-DD-RBF and the 5C4 antibody was 4.78E-11 (Supplementary Fig. 1), which was lower than that for RBF and the 5C4 antibody (1.26E-09). The KD (M) for Post-RBF and 5C4 was 1.27E-07 (Supplementary Fig. 1). The OD450 measured in sandwich ELISA shown that CTA1-DD-RBF and 5C4 antibody have better binding ability, compared with RBF or post-RBF (Supplementary Fig. 2). These results suggested that CTA1-DD-RBF could bind the 5C4 antibody with a higher affinity than RBF and Post-RBF, which indicated that the CTA1-DD-RBF protein exists in the prefusion conformation. Negative stain electron microscopy of the CTA1-DD-RBF protein showed that the protein was highly homogenous (Fig. 1C).

### CTA1-DD-RBF vaccination induced specific humoral immune responses and neutralizing antibodies

**Figure 2:**
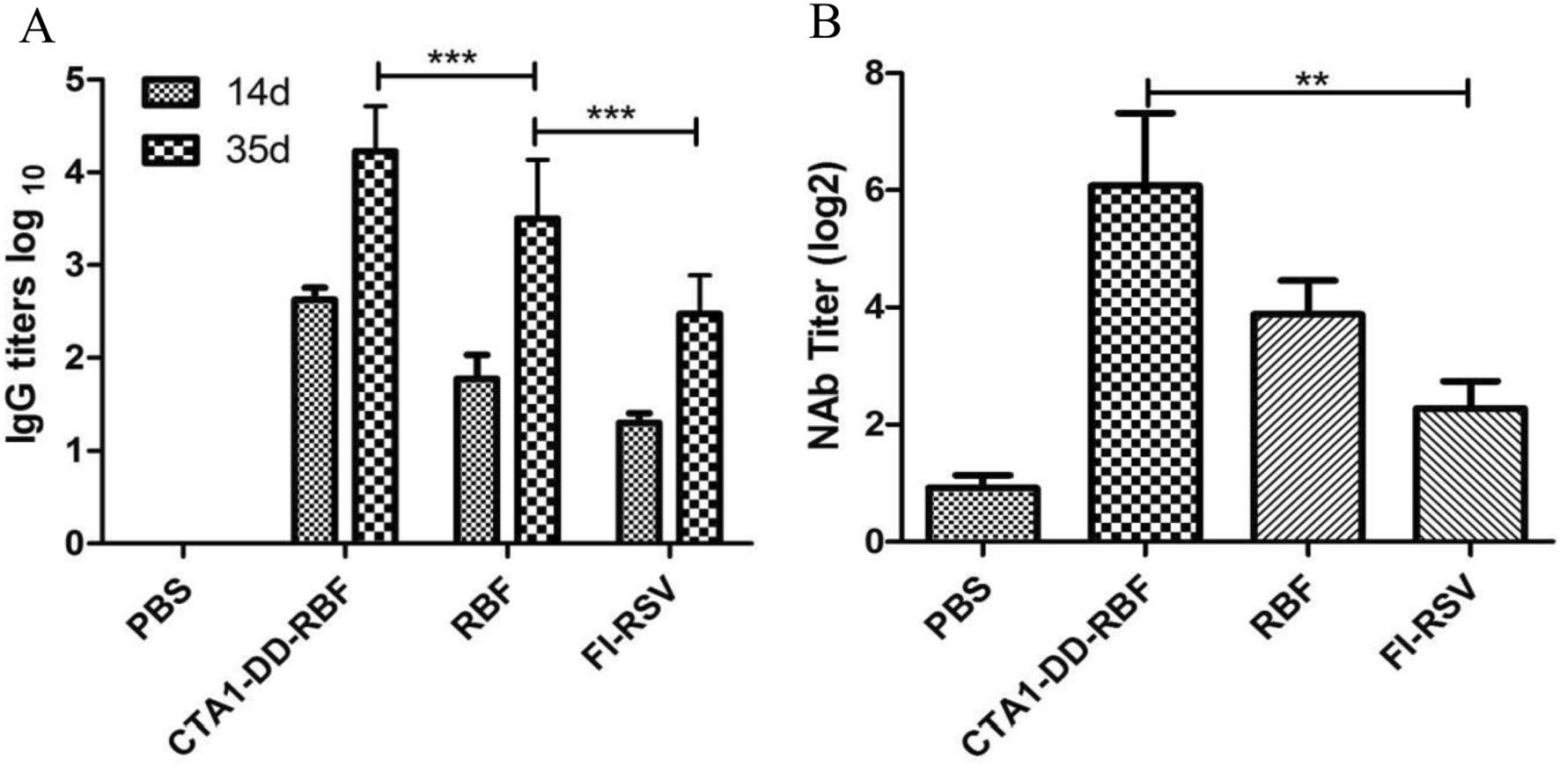
IgG and neutralizing antibodies induced by intranasal vaccination in mice. (**A**) Specific humoral immunity on day 14 (14 days after the initial immunization) and day 35 (14 days after the immunization boost). (**B**) The serum neutralizing titers against hRSV A Long were measured at day 35. The titers are presented as the dilution factors resulting in a 50% reduction in plaque numbers. Statistically significant differences were appropriately determined by one-way ANOVA, ***p < 0.001. The lines represent the median and interquartile range, and the bars represent the mean values with standard deviations.

We evaluated the hRSV-specific humoral immune response by indirect ELISA on day 14 (14 days after the initial immunization) and day 35 (14 days after the immunization boost). Mice immunized with CTA1-DD-RBF exhibited a stronger humoral immune response than mice immunized with the RBF protein alone (Fig. 2A). We also found significant increases in antibody titers at day 35 compared with those at day 14.

The plaque reduction neutralization test showed that the neutralization activity of serum from FI-RSV- and RBF-immunized mice was lower than that of serum from CTA1-DD-RBF-immunized mice (Fig. 2B).

### CTA1-DD-RBF reduced hRSV replication and induced mucosal immune responses

**Figure 3:**
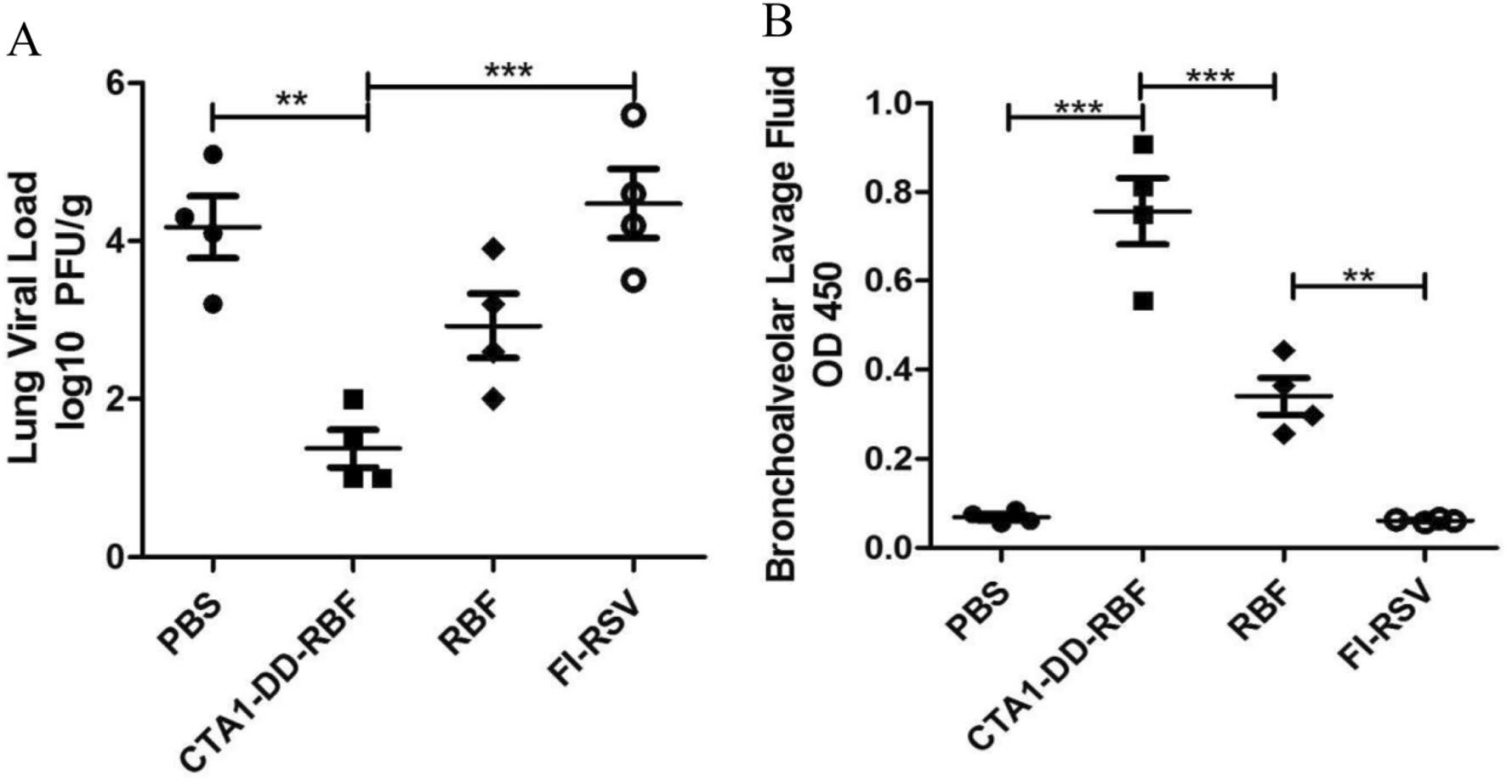
CTA1-DD-RBF reduced hRSV replication and induced neutralizing antibodies in mice. (**A**) The lung viral load was measured on day 4 after hRSV A Long challenge. Each data point indicates a mouse. (**B**) IgA in the bronchoalveolar lavage fluid was detected using ELISA with RBF (100 ng/well) as the coating antigen. Statistically significant differences were measured by one-way ANOVA with the Newman-Keuls posttest, ***p < 0.001. The bars represent the mean values with standard deviations.

All mice were challenged with 1 × 10^5^ PFU of hRSV A Long 14 days after the booster immunization, and the hRSV titers in the lungs were detected at 4 days after hRSV infection. PBS- and FI-RSV-vaccinated mice had substantial amounts of hRSV in their lungs, with values reaching nearly 10^5^ PFU/g. In contrast, CTA1-DD-RBF vaccination reduced hRSV replication and dramatically decreased the viral titer in the lungs. As shown in Fig. 3A, compared with FI-RSV immunization, intranasal immunization significantly reduced the viral load after hRSV infection (p < 0.05).

At 14 days after the second immunization, sIgA in the BALF was detected by ELISA. In the CTA1-DD-RBF group, the BALF sIgA antibody levels were high and were significantly higher than those in the RBF group (Fig. 3B) (p < 0.005). Taken together, these results indicated that CTA1-DD-RBF promoted the production of sIgA and that intranasal vaccination induced specific humoral, cellular and mucosal immune responses.

### CTA1-DD-RBF stimulates a Th1 immune response

**Figure 4:**
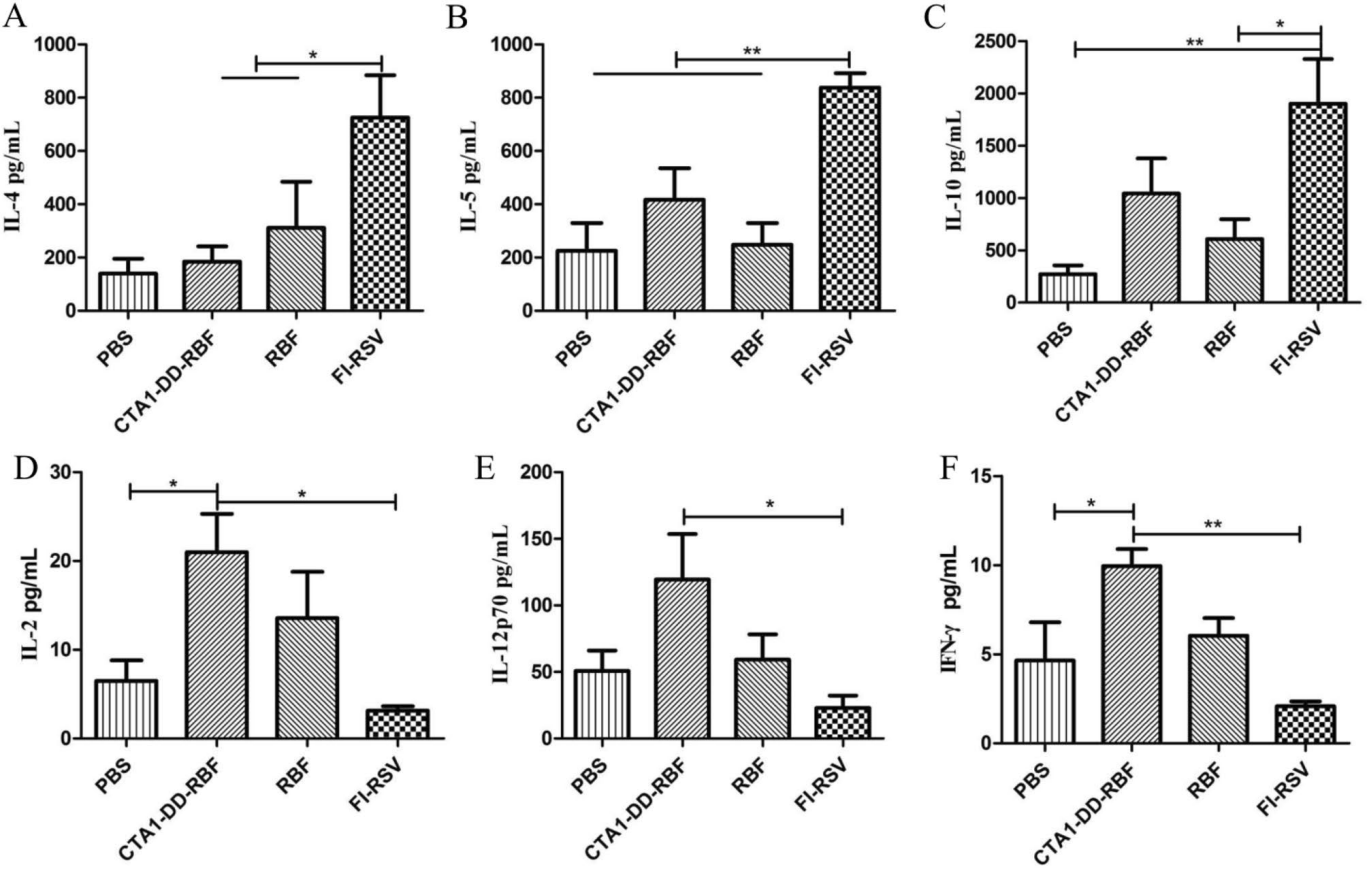
Intranasal vaccination elicited a Th1 cytokine response in bronchoalveolar lavage fluid (BALF). BALF was collected at 14 days after the second immunization. We assessed the contents of Th1-type cytokines (IL-2, IL-12p70, IFN-γ) and Th2-type cytokines (IL-10, IL-4, IL-5) in BALF using ELISA kits (Dakewei, Beijing, China) according to the manufacturer's instructions. Statistically significant differences were determined by one-way ANOVA with the Newman-Keuls posttest. *p < 0.05, **p < 0.01, ***p < 0.001. The bars represent the mean values with standard deviations.

To assess the type of immune response induced by intranasal vaccination, we assessed the contents of Th1-type cytokines (IL-2, IL-12p70, IFN-γ) and Th2-type cytokines (IL-10, IL-4, IL-5) in BALF using ELISA kits (Dakewei, Beijing, China) according to the manufacturer's instructions. Compared with the FI-RSV group, intranasal vaccination with CTA1-DD-RBF and RBF elicited a Th1 cytokine response in BALF (Fig. 4).

**Figure 5:**
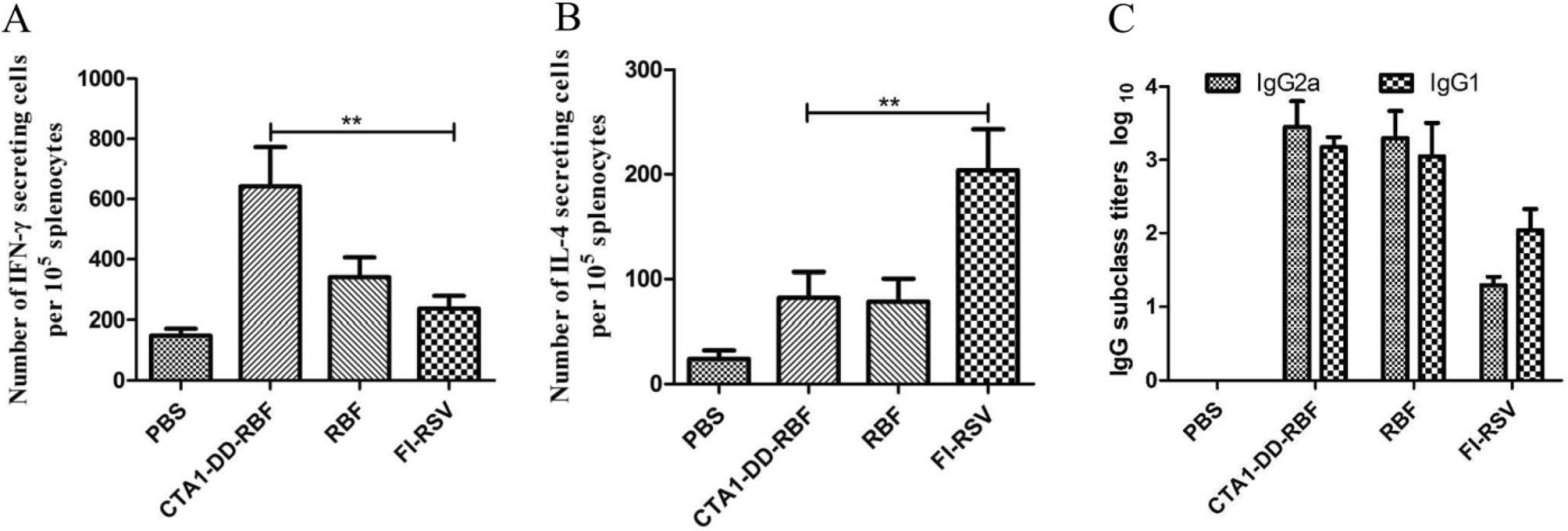
Intranasal vaccination elicited Th1 cellular and humoral immune responses. (**A**, **B**) ELISPOT assays were used to evaluate the type of cellular immune response (Th1 or Th2). The numbers of IL-4- and IFN-γ-secreting cells among spleen cells stimulated with F were determined. (**C**) The titers of IgG1 and IgG2a in serum collected 14 days after the immunization boost were calculated to determine the type of humoral immune response. Statistically significant differences were determined by one-way ANOVA with the Newman–Keuls posttest. *p < 0.05, **p < 0.01, ***p < 0.001. The bars represent the mean values with standard deviations.

To evaluate the type of cellular immune response (Th1 or Th2), we investigated the numbers of IL-4- and IFN-γ-secreting cells among spleen cells stimulated with RBF. The number of IL-4-secreting cells in the FI-RSV group was higher than those in the CTA1-DD-RBF and RBF groups (p < 0.005) (Fig. 5B). Furthermore, high numbers of IFN-γ-secreting cells were induced in the CTA1-DD-RBF group (Fig. 5A).

To further assess the immune response, we investigated the titers of IgG1 and IgG2a in serum collected 14 days after the immunization boost. Compared to the FI-RSV group, the CTA1-DD-RBF and RBF groups exhibited a relatively balanced humoral immune response (Fig. 5C). In conclusion, intranasal vaccination induced a Th1-biased immune response, in contrast to the FI-RSV vaccine.

### CTA1-DD-RBF vaccination reduced lung pathology

**Figure 6:**
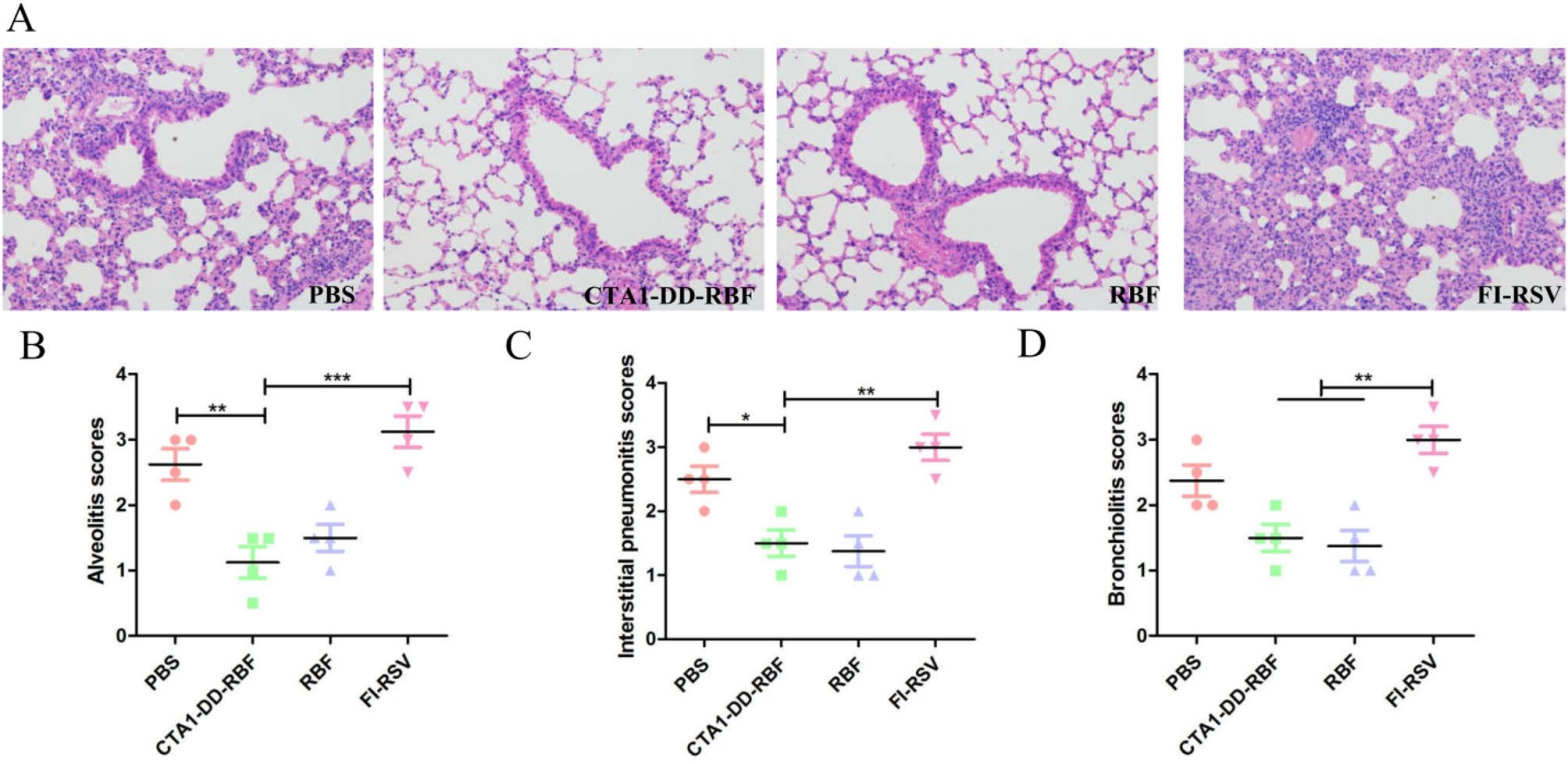
Histopathological analysis of hematoxylin and eosin (H&E)-stained lungs. The left lungs were dyed with H&E for histological assessment. All images were obtained at 200 × magnification. (**A**) The left lungs were dyed with H&E for histological assessment. (**B**) Scoring of alveolitis in immunized mice after hRSV challenge. (**C**) Scoring of interstitial pneumonitis in immunized mice after hRSV challenge. (**D**) Scoring of bronchiolitis in immunized mice after hRSV challenge. The degree of inflammation in the alveolar tissue was graded as follows: 0, normal; 1, mild inflammation; 2, moderate inflammation; 3, marked inflammation; and 4, severe inflammation. Statistically significant differences were determined by one-way ANOVA with the Newman-Keuls posttest. *p < 0.05, **p < 0.01, ***p < 0.001. The bars represent the mean values with standard deviations.

As shown in Fig. 6, after hRSV challenge, mice immunized with FI-RSV had severe alveolitis, interstitial pneumonitis and bronchiolitis (p < 0.001). In contrast, we observed only slight lung infiltration in CTA1-DD-RBF- and RBF-immunized mice. These results demonstrate that intranasal vaccination reduces lung injury after hRSV infection and does not enhance disease in mice.

## Discussion

Because hRSV infects via the respiratory tract, vaccines administered nasally may offer more protective benefits than those delivered intramuscularly. It is important to select the right adjuvant to tailor the required immune response. CTA1-DD has been shown to enhance immunogenicity when mixed or combined with relatively poor vacinnes delivered mucosally^41,42,45^. While CTA1-DD was previously shown to be nontoxic and to not cause inflammation at the application site^46^, it has not been studied in human clinical trials. Proving the safety of mucosal adjuvants is difficult. For example, a mucosal adjuvant derived from *E. coli* (LTK63) is considered to be safe in macaques, Guinea pigs and mice^47^, but the intranasal administration of LTK63 has been shown to induce transient facial nerve paralysis (Bell’s palsy) in humans^48^. Therefore, more thorough toxicological and immunological analyses may be required before CTA1-DD can be studied in clinical trials.

Numerous vaccine candidates are currently under clinical investigation, including live attenuated vaccines, viral vector vaccines, and subunit vaccines^31,49–51^. Nonreplicating vaccines (subunit or inactivated vaccines) should be avoided in hRSV-naïve young children^52^. Live attenuated vaccines and viral vector vaccines tend to be administered intranasally to children^8^. Enhanced pulmonary histopathology was observed in cotton rats immunized intramuscularly with purified F protein^53^. However, the results might depend on the immune dose^54^ and conformation of the protein^55^. Mice immunized intramuscularly with CTA1-DD-RBF had slightly severe alveolitis, interstitial pneumonitis and bronchiolitis.

Multiple in vitro expression systems, including *Escherichia coli*, insect cells (Sf9) and mammalian cells, have been investigated for production of the F protein or peptide as an immunogenic hRSV subunit vaccine^11,44,56^. This study is the first to report that the recombinant CTA1-DD-RBF protein can be successfully expressed in *E. coli*. The *E. coli* protein expression system is one of the simplest and most cost-effective systems and is suitable for the large-scale production of recombinant proteins^57^. BLI analyses herein showed that the KD (M) for CTA1-DD-RBF and the 5C4 antibody (specific to pre-F) was lower than that for RBF and 5C4 (1.26E-09). These results suggested that CTA1-DD-RBF could bind the 5C4 antibody with higher affinity than the Post-RBF protein.

Cellular immune strength, mucosal IgA and serum neutralizing antibody titers were negatively correlated with disease severity^58^. Intranasal vaccination of mice with CTA1-DD-RBF induced hRSV-specific humoral, cellular and mucosal immune responses. The abilities of CTA1-DD-RBF to induce neutralizing serum antibodies and inhibit virus replication in the lungs were superior to those of RBF. Due to the major role of T cell responses in hRSV clearance^59^, we investigated the number of IFN-γ-secreting cells among spleen cells stimulated with F protein^60^, and intranasal immunization with CTA1-DD-RBF significantly increased the number of IFN-γ-secreting cells.

The ability of vaccines administered via the noninvasive mucosal route to induce mucosal antibody responses in addition to systemic antibody responses has been explored^61^. hRSV infects people through mucosal sites, and the mucosal antibody sIgA provides protection at sites of viral entry^62^. A vaccine that elicits a long-lasting hRSV-specific sIgA response is more protective than one that produces only systemic antibodies^63^. Therefore, we evaluated mucosal sIgA in BALF after the nasal administration of the CTA1-DD-RBF protein. At 14 days after the second immunization, the high antigen-specific sIgA in the CTA1-DD-RBF group was significantly higher than that in the RBF group. These results indicated that intranasal immunization with the mucosal adjuvant CTA1-DD could induce better mucosal sIgA antibodies than that with RBF.

An FI-RSV vaccine tested in the 1960s was shown to lead to ERD upon viral challenge^64^. FI-RSV immunization induces a Th2-biased immune response that results in pulmonary eosinophilia following hRSV challenge in multiple animal models^65^. Therefore, hRSV vaccines should be designed to reduce detrimental Th2-biased immune responses and to induce a Th1-type immune response. The serum titers of IgG1 and IgG2a at 14 days post boost immunization and the numbers of IFN-γ- and IL-4-secreting cells among spleen cells stimulated with RBF demonstrated that CTA1-DD-RBF administered mucosally induced a Th1-biased immune response. To further assess the immune response, we investigated the contents of Th1-type cytokines (IL-2, IL-12p70, IFN-γ) and Th2-type cytokines (IL-10, IL-4, IL-5) in BALF. Compared with the FI-RSV group, CTA1-DD-RBF and RBF administered intranasally elicited a Th1 cytokine response in BALF.

Previous studies have shown that immunization with 5 μg^66^ or 3.2 μg^67^ of FI-RSV prevented virus replication in the lungs at day 4 post challenge. However, in this study, the hRSV titers in the lungs of FI-RSV-immunized mice were not reduced compared with those in the lungs of PBS group mice, and the protective effect of FI-RSV may depend on the vaccine dose^54^. The FI-RSV vaccine is considered to be effective when the hRSV titer in the cell culture supernatant is 2 × 10^6^ PFU/ml^68^ or 4.6 × 10^5^ PFU/ml^69^ prior to formalin inactivation. However, the hRSV titer was only 6.5 × 10^4^ PFU/ml prior to formalin inactivation in this study.

These studies have several limitations. First, assessment of cytokines in the BALF is not a great measure of local cellular immune responses in the lung. These limitations will be addressed by our future animal experiments, by flow cytometry after antigen stimulation and intracellular cytokine staining. Second, some measure of memory B and T cells, including local tissue-resident memory T cells in the lung would add significantly to the study. Third, Using i.n. immunization, the relative distribution between the respiratory tract and the gastrointestinal tract is heavily influenced by delivery volume and level of anesthesia^70^. 100 µl of the vaccine is a very high volume and is likely to result in pulmonary and maybe gastric delivery of the antigen in mice, especially under isoflurane anaesthesia. We should reduce protein immune volume in future animal experiments. Fourth, the viral load in the lungs is assessed at one time point post challenge. This would be more convincing if assessed at several time points.

In conclusion, we established a highly efficient prokaryotic expression system for CTA1-DD-RBF protein production that has great potential for expressing hRSV vaccines. Compared with RBF, CTA1-DD-RBF induced higher levels of neutralizing serum antibodies in immunized mice. Compared to FI-RSV mice, mice immunized with CTA1-DD-RBF had no signs of ERD upon hRSV challenge. This study provides insight into developing CTA1-DD-RBF as an effective and safe hRSV mucosal vaccine.

## Materials and methods

### Ethics statement

Female BALB/c mice (6–8 weeks) were purchased from SPF Biotechnology (Beijing). All the protocols were performed in accordance with the guidelines of the Animal Experimental Ethics Committee of the National Institute for Viral Disease Control and Prevention (no. 20190610024). This study was carried out in compliance with the ARRIVE guidelines. The animals were housed under pathogen-free and temperature-controlled conditions at the Animal Center of the Chinese Center for Disease Control and Prevention. The mice were observed every day. The animals were lightly anesthetized with isoflurane for immunizations and blood draws and euthanized with carbon dioxide for terminal organ harvests. All methods were carried out in accordance with relevant guidelines and regulations.

### Construction of expression plasmids

The RBF protein contains a sequence encoding hRSV F residues 26–105 and 146–513 (replacing two furin cleavage sites and P27 with a variable linker, GSGSG) and a T4 fibritin trimerization motif (foldon) at the C-terminus. To retain prefusion-specific neutralizing epitopes and increase the antigenic stability to heat inactivation, DS-Cav1 mutations (S155C and S290C, S190F, V207L) and a disulfide bond (A149C, Y458C) were incorporated into the sequence^71^. We expressed the pre- and postfusion F proteins, called RBF and Post-RBF, respectively, using *Escherichia coli* BL21^72^. The recombinant protein CTA1-DD-RBF contains a CTA1-DD mucosal adjuvant and the RBF protein. CTA1-DD comprises the CTA1 subunit of cholera toxin (CT) and two immunoglobulin (Ig) binding domains (DD) of staphylococcal protein A (DD, 111 aa). The amino acid sequence of the foldon at the C-terminus is GYIPEAPRDGQAYVRKDGEWVLLSTFL. The CTA1-DD-RBF gene was synthesized by Sangon Biological Co., Ltd. (Shanghai, China) and subcloned into the expression vector pET28a between the EcoRI and NotI sites (pET28a-CTA1-DD-RBF) (Fig. 1). The recombinant plasmids were transformed into *E. coli* DH5α and identified by restriction analysis and sequencing.

### Recombinant protein purification and assessment

*E. coli* BL21 (DE3) cells were transformed with the pET28a-CTA1-DD-RBF expression plasmid, and the proteins were induced with 0.1 mM IPTG and expressed as inclusion bodies. The inclusion bodies were dissolved with 8 M urea buffer. After renaturation using various concentrations of urea buffer (8 M–6 M–4 M–2 M–0 M), CTA1-DD-RBF was purified using HisTrap FF columns. The 6 × His-tags of CTA1-DD-RBF were removed by overnight digestion with thrombin at 26 °C, and the proteins were further purified using HisTrap FF column. Before immunization, CTA1-DD-RBF and RBF were purified using Superdex 200 column (GE Healthcare) and concentrated by ultrafiltration using Millipore 15 ml/30KD. The endotoxin concentration was measured with ToxinSensorTM Chromogenic LAL Endotoxin Assay Kit (Genscript).

The binding kinetics of CTA1-DD-RBF, RBF and Post-RBF to the 5C4 antibody (specific to pre-F) were detected using biolayer interferometry (BLI) with the Octet RED96e instrument. The specific method is provided in the methods section of the Supplementary Materials. The RBF and Post-RBF proteins were kept in our laboratory and are described in the Supplementary Material.

### Immunization and challenge

The mice were randomly distributed into experimental groups (8 mice/group) and were vaccinated intranasally or intramuscularly twice with a 3-week interval (Table 1). Briefly, the two groups of mice were vaccinated intranasally with 10 μg of CTA1-DD-RBF or RBF without adjuvant. One group of mice was administered 100 μl of phosphate buffered saline (PBS) twice as a placebo control. To appraise the safety of the mucosal vaccine, we immunized 8 mice with 100 μl of the FI-RSV vaccine via the intramuscular route. FI-RSV was constructed as described by Kim et al.^64^. In brief, 100 mL of clarified hRSV was incubated with formalin (37–40%) at 4,000:1 (v/v) for 72 h at 37 °C and then centrifuged at 50,000×*g* for 1 h at 4 °C. The pellet was diluted in 5 mL of MEM and subsequently mixed with alum adjuvant (4 mg/mL) at 10:1 (v/v). The alum-adsorbed FI-RSV was collected by centrifugation at 1,000 rpm for 10 min, suspended in 1 mL of serum-free MEM, and stored at 4 °C. The mice were infected intranasally with hRSV A Long (1 × 10^5^ PFU) in 100 μl under isoflurane on day 35. Serum samples were collected by centrifugation and kept at − 80 °C until use. For the collection of bronchoalveolar lavage fluid (BALF) and splenocytes, four mice from each group were sacrificed and blend thoroughly on day 35, while the other mice were sacrificed 4 days after the hRSV challenge.

**Table 1:** Animal grouping and immunizations.

Group	Dose	Adjuvant	Route	Immunization days	N
RBF	10 μg	–	i.n	0, 21	8
CTA1-DD-RBF	10 μg	–	i.n	0, 21	8
FI-RSV	1:100 in MEM	Al(OH)3	i.m	0, 21	8
PBS	50 μL	–	i.n	0, 21	8

### Viral detection in the lungs and determination of antibody titers

The right lungs were removed from the mice aseptically at 4 days after the hRSV challenge. The titers of hRSV in the lungs were determined by the plaque test, and the serum neutralizing antibodies were detected by the plaque reduction neutralization test described by Garg et al.^73^. hRSV-specific antibodies (IgG, IgG2a and IgG1) were determined by ELISA using RBF (100 ng/well) as the coating antigen. Endpoint titers were calculated as the highest serum dilution that gave an optical density exceeding 2.1 times the background.

### Detection of cytokines and sIgA in bronchoalveolar lavage fluid

BALF was collected by gentle injection of 1 ml of PBS into the lungs via the trachea with a syringe connected to the trachea 14 days after the second immunization. The hRSV-specific sIgA antibody titer in BALF was determined by ELISA using RBF protein (100 ng/well) as the coating antigen. BALF was added to 96-well plates (100 μl/well) for 1 h, and HRP-conjugated goat anti-mouse sIgA was then diluted 5000 times and incubated with the sample for 1 h; the OD450 was measured using a microplate reader. The BALF levels of Th1-type cytokines (IL-2, IL-12p70, IFN-γ) and Th2-type cytokines (IL-10, IL-4, IL-5) were determined using ELISA kits (Dakewei, Beijing, China) according to the manufacturer's instructions.

### ELISPOT assays

ELISPOT assays were used to evaluate the type of cellular immune response (Th1 or Th2). Splenocytes were collected 14 days after the second immunization according to the manufacturer’s instructions (Dakewe, Beijing, China). To evaluate the cellular immune responses, splenocytes were stimulated with the RBF protein (5 µg/mL).

### Lung histopathology

The left lungs were fixed in 10% neutral buffered formalin at 4 days after the hRSV challenge and stained with hematoxylin and eosin (H&E) for histological evaluation under a Nikon Eclipse light microscope by a board-certified pathologist. Slides stained with H&E were evaluated for the following three parameters described by our previous study: bronchiolitis, alveolitis and interstitial pneumonitis^72^. The slides were evaluated in a blinded fashion using a 1–4 scoring system as follows: 0 (normal), 1 (mild inflammation), 2 (moderate inflammation), 3 (marked inflammation) and 4 (severe inflammation).

### Statistical analysis

All data were analyzed using GraphPad Prism version 5. Differences among all groups were evaluated by one-way ANOVA, followed by a Newman-Keuls posttest. Differences were considered significant if P < 0.05.

## Supplementary Information

Supplementary Information.

## Data Availability

All data included in this study is available upon request by contact with the corresponding author.
