# Supplementary material for: Intranasal vaccination with a recombinant protein CTA1-DD-RBF protects mice against hRSV infection
Source: Sci Rep. 2021 Sep 20;11:18641. doi: 10.1038/s41598-021-97535-6 (PMC8452643; doi:10.1038/s41598-021-97535-6)
Supplement: Supplementary file 1 — Supplementary Information. [file 41598_2021_97535_MOESM1_ESM.docx]

**Supporting Information**

**Binding assay**

Binding kinetics of the CTA1-DD-RBF, RBF and Post-RBF to 5C4 antibody were detected using Bio-Layer Interferometry (BLI) with Octet RED96e. We already described the RBF and Post-RBF in the article “hRSV F protein expressed in Pichia pastoris or Escherichia coli induces protective immunity without inducing enhanced respiratory disease in mice” published in Archives of Virology.

CTA1-DD-RBF was mixed with polyclonal IgG prior to detection. The final volume for all solutions was 200 μl/well. Assays were performed at 30 °C in solid black 96-well plates. Data analysis and curve fitting were carried out using Octet software.


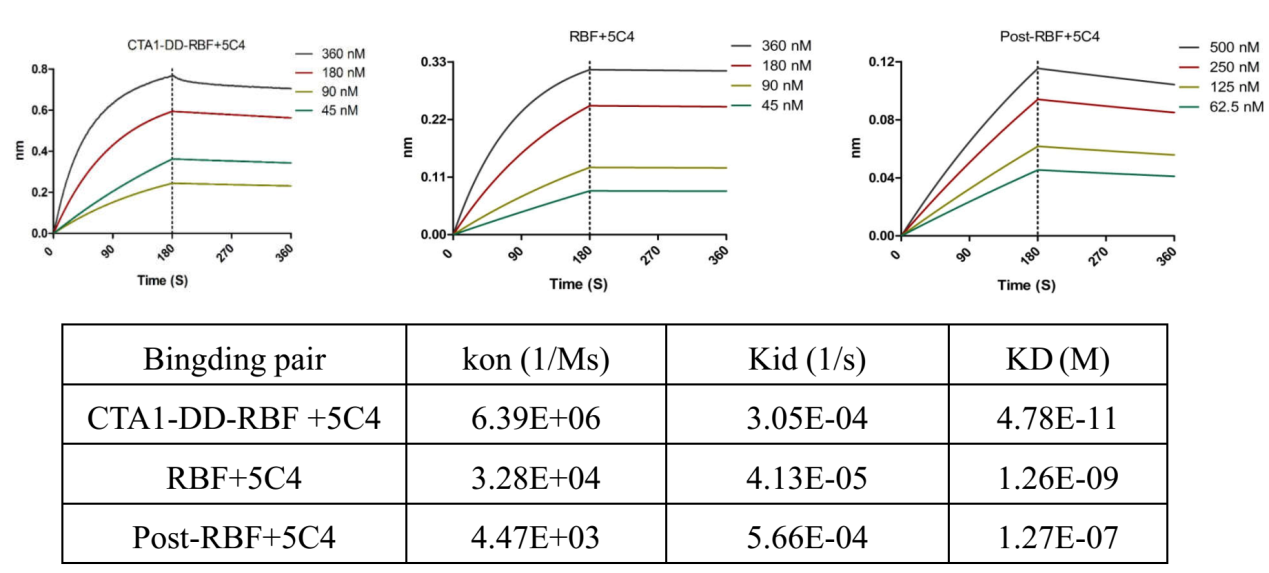


Supplementary Fig. 1. Kinetics of binding of CTA1-DD-RBF, RBF and Post-RBF to the pre-F-specific antibody 5C4. Kon is the association constant. Kid is the dissociation constant. KD is the ratio of Kid to Kon, representing the affinity constant.

**Sandwich ELISA**

The binding capabilities of 5C4 with CTA1-DD-RBF, RBF or post-RBF were determined by sandwich ELISA as described by Kirsten et al. ^(Kim et al., 1969)^ with a slight modifications. The monoclonal antibody 5C4 (specific to pre-F) was used to coat 96 plates as capture antibody at 200 ng/well. The proteins were diluted to 0.001μg/mL, 0.01μg/mL, 0.1μg/mL, 1μg/mL and 10μg/mL. Add 100μL to each well and incubate at room temperature for 60 minutes. Then palivizumab and anti-human IgG conjugated with HRP were used to detect bound antigens. The OD450 was measured using a microplate reader.


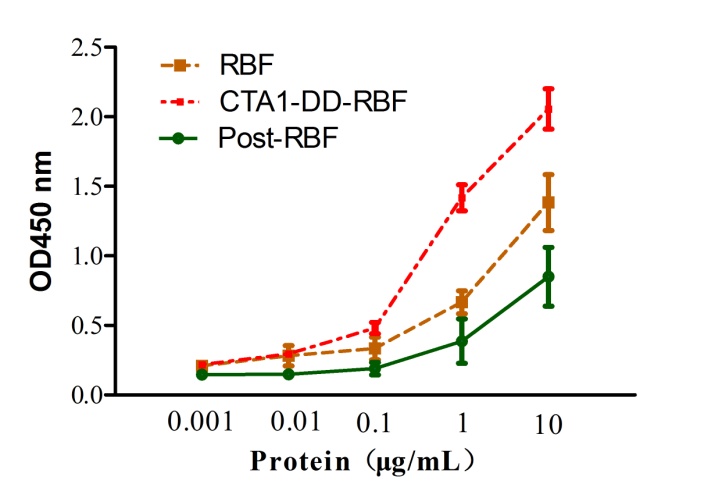


Supplementary Fig. 2. The binding capabilities of 5C4 with CTA1-DD-RBF, RBF or post-RBF were assessed by sandwich ELISA.

**CTA1-DD-RBF proteins on SDS-PAGE**


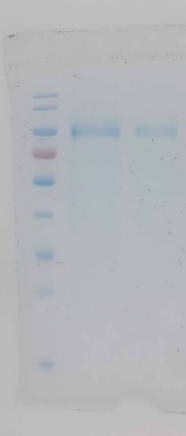


Kim, H.W., Canchola, J.G., Brandt, C.D., Pyles, G., Chanock, R.M., Jensen, K., Parrott, R.H., 1969. Respiratory syncytial virus disease in infants despite prior administration of antigenic inactivated vaccine. Am J Epidemiol 89, 422-434.
